# A retrospective outcomes study 25-gauge 10,000 CPM beveled-tip and 25-gauge flat-tip microincision vitrectomy for proliferative diabetic retinopathy treatment

**DOI:** 10.3389/fmed.2025.1614668

**Published:** 2025-08-26

**Authors:** Guangjie Han, Jianwei Zhai, Hongbo Huang, Limei He, Heruo Wei, Lirong Wei, Huanyan Wang

**Affiliations:** Department of Ophthalmology, Liuzhou Red Cross Hospital, Eye Hospital of Liuzhou City, Liuzhou, Guangxi, China

**Keywords:** vitrectomy, proliferative diabetic retinopathy, vitreoretinal surgery, 25G flat-tip MIVS, 25G 10K cpm beveled-tip MIVS

## Abstract

**Background:**

To evaluate the therapeutic efficacy and safety of 25-gauge (25G) 10,000 cpm (10K) beveled-tip microincision vitrectomy (MIVS) versus 25-gauge (25G) flat-tip MIVS in managing proliferative diabetic retinopathy (PDR).

**Methods:**

This retrospective study involved 60 eyes with proliferative diabetic retinopathy (PDR) from 60 patients, all requiring epiretinal membrane removal. The patients were assigned to either the 25G 10K cpm beveled-tip MIVS group or the 25G flat-tip MIVS group. Surgical outcomes, including membrane removal efficiency, vitrectomy probe (VP) and microforceps exchanges, total procedure duration, vitrectomy time, and intraoperative complications, were documented. Best-corrected visual acuity (BCVA), intraocular pressure (IOP), and postoperative complications were assessed during a 6-month follow-up period.

**Results:**

Fifty-eight eyes (from 58 patients) completed follow-up, including 30 eyes in the 25G 10K cpm beveled-tip group and 28 eyes in the 25G flat-tip group. During surgery, the 25G 10k cpm beveled-tip group demonstrated more effective membrane cutting (*p* = 0.001) and required fewer exchanges between the vitrectomy probe and microforceps (*p* = 0.001). The total surgery time and vitrectomy time were both reduced in this group (*p* = 0.001 and *p* = 0.001, respectively). Additionally, fewer intraoperative hemostasis maneuvers were needed in the 25G 10K cpm beveled-tip group. All follow-up outcomes indicated no significant differences between the two groups.

**Conclusion:**

In the surgical treatment of PDR, the 25G 10K cpm beveled-tip MIVS group showed no statistically significant difference compared to conventional 25G flat-tip MIVS in terms of visual acuity improvement and postoperative intraocular pressure. However, the former demonstrated advantages such as reduced surgical time, decreased intraoperative use of electrocoagulation, and fewer instrument exchanges within the eye, providing robust evidence for its efficacy in PDR surgical management.

## Introduction

Diabetic retinopathy (DR) is a leading cause of blindness, posing a significant threat, particularly to working-age populations (1). The global prevalence of diabetic retinopathy is increasing. Systematic evaluations indicate it is significant worldwide and even higher in regions such as Africa and the Middle East (2). DR is classified into non-proliferative and proliferative (PDR) types, with PDR being more severe due to neovascular rupture, which can lead to vitreous hemorrhage and tractional retinal detachment, resulting in complex treatment and significant visual impairment (3). Pars plana vitrectomy (PPV) is the primary treatment for PDR (4). Since Machemer first reported the 17G three-port PPV in 1971, the technique has been in use for over 50 years (5). In 1972, the 20G PPV was developed as a smaller incision alternative, followed by continuous advancements in minimally invasive techniques (6). The introduction of the 25G sutureless microincision PPV in 2002 reduced incision size to 0.5 mm, simplifying procedures and lowering postoperative complications, though early devices lacked rigidity (7). In 2005, Eckardt refined the 23G PPV, and in 2010, Oshima introduced the 27G system, further enhancing minimally invasive outcomes (8). In recent years, cutting rates in PPV have reached 20,000 CPM, significantly improving surgical stability and efficiency, representing a comprehensive integration of “minimally invasive” concepts, techniques, and equipment.

Minimally invasive vitreoretinal surgery (MIVS) for PDR aims to restore retinal attachment by clearing vitreous hemorrhage and relieving traction, thereby improving visual prognosis (9). Advances in technology have enabled more precise and efficient surgeries for complex tractional retinal detachment. Modern vitrectomy cutters feature beveled designs, which increase the cutter radius and allow closer proximity to tissue surfaces, enhancing surgical control and precision while facilitating the application of the “shovel cutting” technique (10). This design shortens the distance between the cutting plane and the tissue, enabling easier separation and removal of proliferative membranes, reducing instrument exchanges, and improving efficiency. The high cutting speed 10K (10,000 cpm) of beveled cutters further minimizes retinal traction and reduces complications, enhancing safety and precision. 25G 10K cpm beveled-tip cutter system used to treat various vitreoretinal diseases, including vitreomacular traction syndrome, macular holes, epiretinal membranes, rhegmatogenous retinal detachment, and PDR, achieving satisfactory cutting efficiency and shorter surgical durations (10). These advancements appear beneficial for vitrectomy procedures requiring delicate and complex maneuvers. However, despite the availability of advanced vitrectomy technologies, research specific to PDR treatment remains limited. Therefore, this study aims to compare the effectiveness and safety of 25G 10K cpm beveled-tip MIVS with conventional 25G flat-tip MIVS in the treatment of PDR.

## Methods

### Ethics approval and consent to participate

Ethical approval was obtained from the Ethics Committee of (2024.NO.003), and all patients signed informed consent forms of participants involved.

### Participants

Inclusion criteria: (1) Patients diagnosed with PDR confirmed through examination with a 90D lens or a three-mirror lens, supplemented by ophthalmic B-scan ultrasonography. (2) Patients requiring surgical intervention, with the decision on preoperative intravitreal anti-VEGF drug pretreatment 3–5 days before surgery based on clinical condition and financial considerations. (3) Patients able to comply with postoperative follow-up as required for at least 6 months. Exclusion criteria: (1) Presence of rhegmatogenous retinal detachment. (2) Presence of choroidal detachment. (3) History of prior vitrectomy or glaucoma surgery. (4) Patients with severe systemic diseases or those unable to maintain the required surgical position. Based on the inclusion and exclusion criteria, 60 eligible patients (60 eyes) were selected (if both eyes met the criteria, the right eye was included). Randomization was performed using a random number table. Due to the nature of the surgery, blinding was not feasible for the surgeon; however, postoperative follow-up personnel were blinded and were not informed of the specific surgical procedure employed.

### Procedures

All patients provided signed informed consent after being thoroughly informed prior to surgery. The decision to administer preoperative intravitreal anti-VEGF drug pretreatment 3–5 days before surgery was made based on the clinical condition and financial situation of each patient. The surgical equipment used in this study included the Alcon Constellation vitrectomy system. Surgeries were performed under a Zeiss Lumera 700 surgical microscope with a Resight 700 non-contact wide-angle viewing system. All procedures were conducted by the same experienced associate chief ophthalmologist.

25G 10K cpm beveled-tip MIVS was performed under local retrobulbar anesthesia. Preoperative evaluation determined the necessity of cataract surgery, with phacoemulsification to remove the opacified lens, and the decision for primary intraocular lens implantation was based on the patient’s fundus condition. Conjunctival displacement of 2–3 mm was performed at 3.5–4.0 mm posterior to the limbus at the inferotemporal, superotemporal, and superonasal positions, creating conventional scleral ports with cannulas. The inferotemporal port was used for intraocular infusion, while the other two ports were used for fiber optic illumination and intraocular instrument operation by the surgeon. The vitrectomy cutter operated at a speed of 10,000 cuts per minute with a negative pressure of 450 mmHg, maintaining an intraoperative infusion pressure of 28 mmHg. The procedure utilized a 10K 25G beveled vitrectomy pack. Under triamcinolone staining, vitreous hemorrhage and cortical vitreous were meticulously removed, accompanied by scleral indentation to excise the peripheral vitreous base. For membrane peeling, the vitrectomy probe or retinal forceps were used single-handedly to separate proliferative membranes, with tightly adhered membranes being segmented into islands using the probe for gradual removal. In cases of recurrent bleeding from neovascularization, intraocular cauterization was employed for hemostasis. Subretinal fluid was drained when present to ensure retinal reattachment, followed by laser sealing of retinal tears and panretinal photocoagulation. The procedure concluded with the infusion of balanced saline solution, silicone oil, inert gas, or sterile air as required. The scleral cannulas were then removed, and the scleral incision sites were checked for leakage, with sutures applied if necessary.

25G flat-tip MIVS was performed under local retrobulbar anesthesia. Preoperative evaluation determined the necessity for cataract surgery, involving phacoemulsification to remove the opacified lens, with the decision for primary intraocular lens implantation based on the patient’s fundus condition. Conjunctival displacement of 2–3 mm was performed at 3.5–4.0 mm posterior to the limbus at the inferotemporal, superotemporal, and superonasal positions, creating conventional scleral ports with cannulas. The inferotemporal port was used for intraocular infusion, while the other two ports were utilized for fiber optic illumination and intraocular instrument operation by the surgeon. The vitrectomy cutter operated at a speed of 5,000 cuts per minute with a negative pressure of 450 mmHg, maintaining an intraoperative infusion pressure of 28 mmHg. The procedure used a 25G flat-ended vitrectomy pack. Vitreous hemorrhage and cortical vitreous were thoroughly removed under triamcinolone staining, with scleral indentation assisting in the excision of the peripheral vitreous base. For membrane peeling, the vitrectomy probe, retinal forceps, or scissors were used single-handedly to detach proliferative membranes. Tightly adhered membranes were segmented into islands using the probe for gradual excision. In cases of repeated bleeding from neovascularization, intraocular cauterization was performed for hemostasis. Subretinal fluid was drained when present to ensure retinal reattachment, followed by laser sealing of retinal tears and panretinal photocoagulation. The procedure concluded with the infusion of balanced saline solution, silicone oil, or sterile air as needed. The scleral cannulas were then removed, and the scleral incision sites were inspected for leakage, with sutures applied if necessary. After surgery, patients were instructed to maintain a prone position for 5–7 days according to the vitreous infusion and were prescribed tobramycin-dexamethasone eye drops, to be used four times daily for 7–10 days. Follow-up evaluations were conducted over a 6-month period, including assessments of visual acuity, intraocular pressure, and any complications.

### Outcomes

Intraoperative parameters included total surgical duration, vitreous cutting time, membrane peeling duration, the number of iatrogenic retinal tears, cases requiring combined cataract surgery, intraoperative bleeding, the frequency of cauterization for hemostasis, and the number of instrument exchanges within the eye. Postoperative outcomes were assessed at 1 day, 7 days, 1 month, 3 months, and 6 months following surgery, with evaluations of best-corrected visual acuity (BCVA), intraocular pressure, retinal thickness measurements with OCT and the incidence of postoperative complications for both groups.

### Statistical analysis

The sample size was calculated based on primary outcomes with a significance level of *α* = 0.05 and a power of 0.8. Assuming membrane cutting rates of 3 optic discs/min for the 25G 10K cpm beveled-tip MIVS group and 2 optic discs/min for the 25G flat-tip MIVS group, with a standard deviation of 1.2 discs/min, 45 eyes were needed. Including a 10% dropout rate, the final sample size was adjusted to 25 participants per group, totaling 50 eyes.

Statistical analysis was conducted using SPSS (IBM SPSS Statistics 25.0). For normally distributed data, confirmed by the Shapiro–Wilk test, results were expressed as mean ± standard deviation and compared using a two-tailed *t*-test. Non-normally distributed data were presented as median (interquartile range) and analyzed using the Kruskal–Wallis test. Categorical variables were expressed as percentages and compared using the chi-square test or Fisher’s exact test. Changes in BCVA and IOP over time were analyzed using generalized estimating equations (GEEs). A *p*-value of <0.05 was considered statistically significant.

## Results

**Figure 1:**
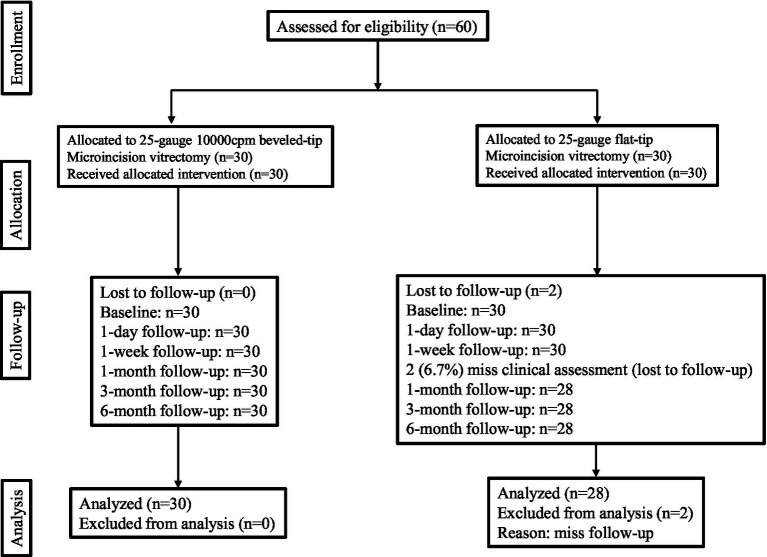
A flow diagram illustrating the retrospective process, follow-up stages, and analysis of the intention-to-treat population.

A total of 60 eyes (60 patients) were enrolled, with 30 eyes assigned to the 25G 10K cpm beveled-tip MIVS group and the remaining 30 to the 25G flat-tip MIVS group. Two patients from the 25G flat-tip MIVS group were lost to follow-up. The study flowchart is shown in Figure 1, and baseline characteristics of both groups are summarized and compared in Table 1. No significant differences were found between the groups regarding age, sex, glycated hemoglobin (HbA1c), duration of diabetes (DM), proliferative membrane grading, macular involvement, number of retinal detachments, lens status, best-corrected visual acuity (BCVA), intraocular pressure, prior anti-VEGF treatment, or preoperative panretinal photocoagulation (PRP).

**Table 1:** Baseline demographics and clinical data of the two groups.

	25G 10K cpm bevel-tip (30)	25G flat-tip (28)	*p*-value
Age (year)	51.6 ± 7.7	54.2 ± 7.5	0.218^*^
Male sex	13	13	0.884^†^
HbA1c (%)	7.84 ± 0.89	7.81 ± 0.82	0.798^*^
Duration of DM (year)	14.8 ± 3.2	14.9 ± 3.1	0.984^‡^
Macula involved	27(90.0%)	26(92.8%)	
Number of retinal detachments	26(86.6%)	25(89.3%)	
Lens status			
Phakic	25(83.3%)	25(89.3%)	
Pseudophakic	5(16.7%)	3(10.7%)	
Aphakic	0	0	
Preop BCVA (logMAR)	1.43 ± 2.8	1.45 ± 0.31	0.976^*^
Preop IOP (mmHg)	16.13 ± 3.18	16.89 ± 3.00	0.854^*^
Number of preop IVI Anti-VEGF treatments	22(73.3%)	20(71.4%)	0.871^†^
Number of preop PRP	3(10.0%)	4(14.3%)	0.658^†^

^*^Based on *t*-test; ^†^based on Chi-square test/Fisher’s exact test; ^‡^based on Mann–Whitney U test.

### Surgical procedure

Table 2 shows the intraoperative data obtained from the surgical videos. The membrane cutting efficiency in the 25G 10K cpm beveled-tip MIVS group was significantly higher than that in the 25G flat-tip MIVS group (*p* = 0.001). Similarly, one of the secondary outcome indicators, the number of times the VP was replaced with micro-forceps, was also significantly lower in the 25G 10K cpm beveled-tip MIVS group (*p* = 0.001). Additionally, the surgery time vitreous removal time in the 25G group was slightly shorter than in the 25G flat-tip MIVS group (*p* = 0.001). In addition, no statistically significant differences were found in other intraoperative indicators, such as concurrent cataract surgery, type of tamponade used, and wound suturing.

**Table 2:** Surgical procedure of the two groups.

	25G 10K cpm bevel-tip (30)	25G flat-tip (28)	*p-*value
Total time (min)	75.83 ± 11.05	80.29 ± 11.82	0.034^*^
Number of VP exchanges to microforceps (n)	4.60 ± 1.14	6.50 ± 1.12	0.001^*^
Productivity of cutting the membrane (optic discs/min)	2.47 ± 0.28	2.17 ± 0.36	0.001^*^
Core vitrectomy time (min)	15.9 ± 0.16	18.6 ± 0.23	0.001^‡^
Number undergoing simultaneous cataract surgery	5 (16.7%)	4 (14.3%)	
Endotamponade substance			
Room air	15 (50.0%)	15 (53.6%)	
Silicone oil	15 (50.0%)	13 (46.4%)	
Number requiring wound sutures	24 (80.0%)	22 (79.6%)	0.999^†^

^*^Based on *t*-test; ^†^based on Chi-square test/Fisher’s exact test; ^‡^based on Mann–Whitney U test.

### Intraoperative and postoperative complications

The comparison of complications between the two groups is detailed in Table 3. No significant differences were observed in the incidence of iatrogenic retinal tears, iatrogenic hemorrhage, the number of electrocoagulations, or the occurrence of cataracts. During the 6-month postoperative follow-up, none of the enrolled patients experienced infectious endophthalmitis or recurrent retinal detachment, and no additional bleeding occurred. Both groups exhibited episodes of increased intraocular pressure (>25 mmHg) and hypotension (<6.5 mmHg) at various time points. In most cases, intraocular pressure returned to baseline following the administration of prescribed eye drops.

**Table 3:** Intraoperative and postoperative complications of the two groups.

	25G 10K cpm bevel-tip (30)	25G flat-tip (28)	*p*-value
Intraoperative complications			
Retinal break (number per operation)	0 (0.1)	1 (0.2)	0.324^†^
Iatrogenic hemorrhage (number per operation)	1 (0.3)	2 (0.4)	0.002
Electrocoagulation (number per operation)	0 (0.1)	1 (0.2)	0.019
Number of iatrogenic cataracts	0	0	
Postoperative complications			
Number of endophthalmitis	0	0	
Number of retinal detachments	0	0	
Number of vitreous hemorrhages			
1 day after surgery	2 (6.7%)	1 (3.6%)	0.255^†^
2–7 days	1 (3.3%)	1 (3.6%)	0.961^†^
2–4 weeks	0	0	
2–3 months	0	0	
3–6 months	0	0	
Number of ocular hypotension			
1 day after surgery	0 (0%)	0 (0%)	
2–7 days	0 (0%)	0 (0%)	
2–4 weeks	0	0	
2–3 months	0	0	
3–6 months	0	0	
Number of ocular hypertension			
1 day after surgery	1 (3.3%)	2 (7.1%)	
2–7 days	1 (3.3%)	1 (3.6%)	0.999
2–4 weeks	0	0	
2–3 months	0	0	
3–6 months	0	0	

^†^Statistical significance was assessed using the Chi-square test or Fisher’s exact test.

### Changes in BCVA and IOP

**Figure 2:**
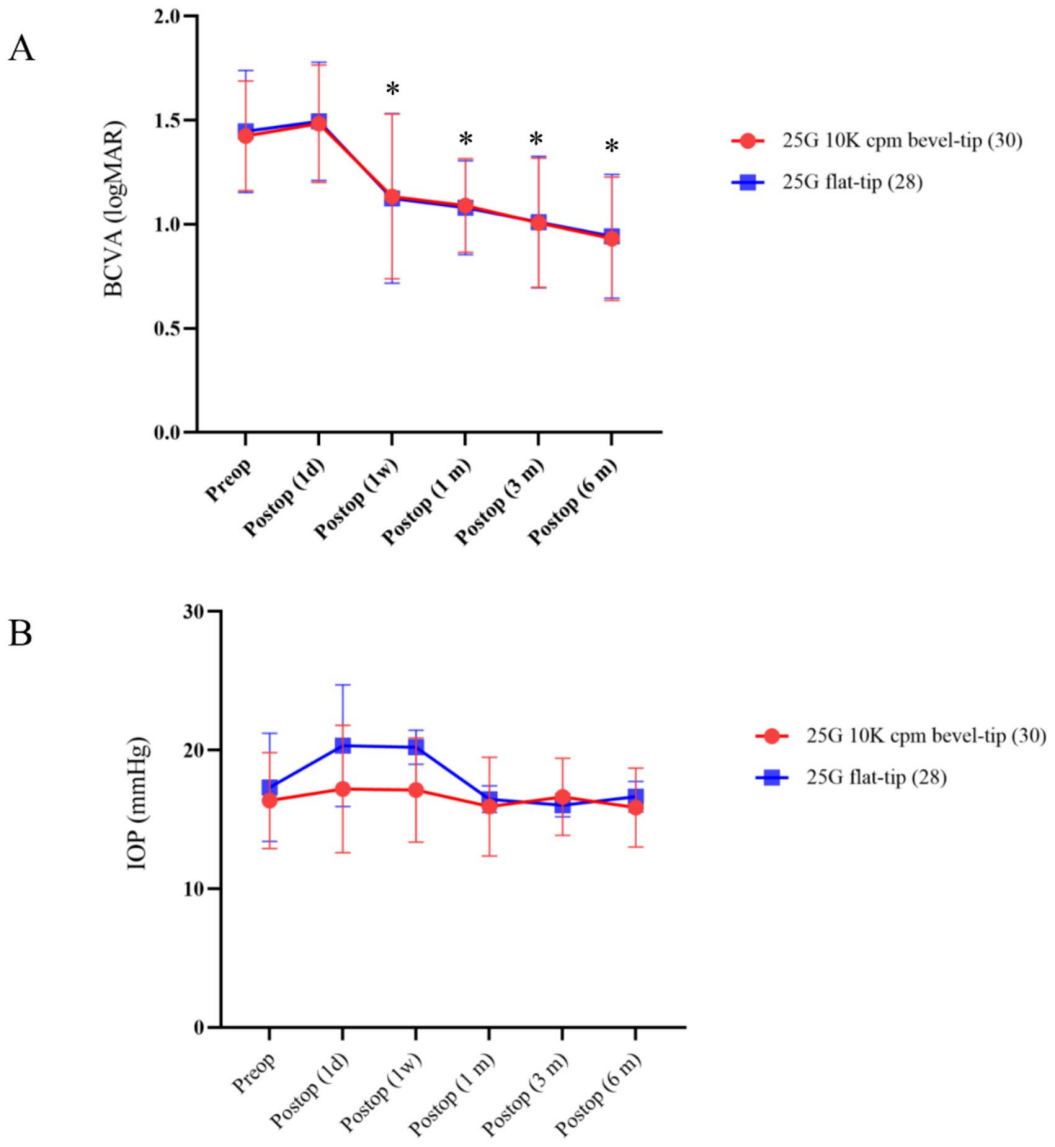
Time course of BCVA and IOP in the two groups. **(A)** Displays the mean BCVA changes over time, from preoperative to 6 months postoperative. **(B)** Shows the mean IOP changes at corresponding time points. *Denotes a statistically significant difference between preoperative and postoperative measurements (*p* < 0.05).

Table 4 and Figure 2 show that both groups experienced significant improvements in best-corrected visual acuity (BCVA) at 1 week, 1 month, 3 months, and 6 months postoperatively, when compared to baseline preoperative values (*p* = 0.011, with *p* < 0.001 for subsequent time points). However, no statistically significant difference was found in the extent of BCVA improvement between the groups. A significant increase in intraocular pressure (IOP) was observed at 1 week postoperatively (*p* = 0.042), but IOP remained within normal ranges at all other time points for both groups. Although IOP rose significantly at 1 week, it returned to normal during subsequent evaluations.

**Table 4:** Changes in BCVA and IOP in the two groups.

	25G 10K cpm bevel-tip (30)	25G flat-tip (28)	*p*-value (Preop *vs.* Postop)
BCVA (logMAR)			
Preop	1.43 ± 0.28	1.45 ± 0.31	
Postop (1d)	1.52 ± 0.27	1.50 ± 0.27	0.768
Postop (1w)	1.11 ± 0.36	1.15 ± 0.30	0.011
Postop (1 m)	1.07 ± 0.23	1.12 ± 0.26	0.001
Postop (3 m)	1.00 ± 0.31	1.10 ± 0.31	0.001
Postop (6 m)	0.92 ± 0.30	1.08 ± 0.28	0.031
*p*-value (25G 10K cpm bevel-tip vs. 25G flat-tip)	0.45		
IOP (mmHg)			
Preop	16.13 ± 3.18	16.89 ± 3.00	
Postop (1w)	16.93 ± 3.71	19.34 ± 5.11	0.042
Postop (1 m)	15.87 ± 3.16	16.39 ± 3.11	0.609
Postop (3 m)	16.13 ± 3.01	16.12 ± 3.08	0.751
Postop (6 m)	16.03 ± 3.10	16.43 ± 3.12	0.634
*p*-value (25G 10K cpm bevel-tip vs. 25G flat-tip)	0.58		
OCT-cut (room air)			
Preop	335 ± 42.4 μm	336 ± 39.3 μm	
Postop (1d)	Not measurable	Not measurable	Not measurable
Postop (1w)	312 ± 35.2 μm	313 ± 34.6 μm	0.001
Postop (1 m)	309 ± 34.1 μm	310 ± 33.4 μm	0.001
Postop (3 m)	306 ± 31.6 μm	304 ± 31.1 μm	0.001
Postop (6 m)	302 ± 30.6 μm	301 ± 30.7 μm	0.001
*p*-value (25G 10K cpm bevel-tip vs. 25G flat-tip)	0.94		
OCT-cut (silicone oil)			
Preop	346 ± 45.0 μm	345 ± 47.2 μm	
Postop (1d)	322 ± 37.5 μm	321 ± 36.1 μm	0.001
Postop (1w)	312 ± 34.7 μm	317 ± 34.8 μm	0.001
Postop (1 m)	308 ± 33.2 μm	313 ± 32.3 μm	0.001
Postop (3 m)	305 ± 31.3 μm	308 ± 30.8 μm	0.001
Postop (6 m)	303 ± 30.3 μm	304 ± 30.2 μm	0.001
*p*-value (25G 10K cpm bevel-tip vs. 25G flat-tip)	0.90		

Group comparisons were conducted using GEE to assess the changes over time.

### Changes in central macular retinal thickness

This dataset compares changes in central foveal retinal thickness following vitrectomy using two 25-gauge vitrectomy probes (10K cpm bevel-tip vs. flat-tip), including analyses of both the standard surgery group and a silicone oil-filled subgroup (Table 4). Retinal thickness at all postoperative time points was significantly reduced compared to preoperative measurements (*p* = 0.001), with no statistically significant difference observed between the two instruments (*p* > 0.90).

## Discussion

PDR represents the advanced stage of DR, characterized by severe complications, such as vitreous hemorrhage and retinal detachment, resulting from the abnormal growth of retinal neovascularization (11). Currently, surgical intervention remains a critical approach to managing these complications. Vitrectomy is the most commonly employed surgical technique, particularly in treating persistent vitreous hemorrhage and retinal detachment caused by neovascular proliferative membranes (12). This procedure restores or preserves vision by clearing opacified vitreous, removing proliferative membranes, and repairing the retina.

With the advent of MIVS, the safety and efficacy of surgical treatment have been significantly enhanced. MIVS utilizes smaller incisions (e.g., 27G or 25G), reducing the need for postoperative sutures, shortening recovery time, alleviating postoperative discomfort, and minimizing corneal astigmatis (13). In recent years, 25G and 27G vitrectomy cutters have increasingly adopted beveled-tip designs, facilitating easier access to subretinal spaces and reducing intraocular tissue damage (14). When the beveled cutter is inserted between membrane layers and advanced, the membrane is lifted and smoothly aspirated into the cutter. This technique, referred to as the “shovel cutting” technique by its designers, has proven to be efficient. Furthermore, the introduction of ultra-high-speed cutting systems, with cutting rates up to 10,000 cpm, has improved surgical efficiency and stability, reducing operative time and the risk of complications (15). Although clinical trials on beveled-tip MIVS remain limited, current evidence suggests its potential advantages in the treatment of PDR.

To evaluate the role of the 25G 10K cpm beveled-tip MIVS in the treatment of advanced PDR, we used the 25G flat-tip cutter (5,000 cpm) as a control group. A comparative analysis was conducted on total surgical time, vitreous cutting time, proliferative membrane handling time, intraoperative instrument exchange frequency, intraoperative hemorrhage and diathermy counts, preoperative and postoperative visual acuity, intraocular pressure changes, and the occurrence of intraoperative and postoperative complications. This study observed that, compared with traditional 25G flat-tip MIVS, the 25G 10K cpm beveled-tip cutter demonstrated superior membrane removal efficiency and significantly fewer exchanges between the vitrectomy probe and microforceps. Surgeons also reported that the beveled-tip cutter avoided many repetitive and ineffective maneuvers commonly encountered with flat-tip cutters, highlighting its versatility and efficiency. These findings suggest that advanced vitreous cutters can enhance membrane removal efficiency while reducing retinal damage caused by frequent instrument exchanges.

According to existing clinical and retrospective studies, the beveled design increases the contact area between the cutter and vitreous, potentially accelerating the cutting speed and improving overall surgical performance (15). The 25G beveled cutter system features ultra-high-speed cutting capabilities (10,000 cpm), designed to maximize vitreous cutting efficiency. One study indicated that the cutting efficiency of the 25G 10K cpm system significantly outperformed conventional 25G systems, with the beveled-tip cutters proving to be markedly more efficient than flat-tip cutters. Our study corroborated these findings, observing that the 25G 10K cpm beveled-tip cutter was superior to the 25G flat-tip cutter (5,000 cpm) in core vitreous removal. We also found that core vitreous removal time significantly impacted overall surgical duration, with the 25G 10K cpm system significantly reducing operative time compared to flat-tip cutters. Notably, prior studies have shown that 27G beveled-tip cutters, due to their smaller diameter, result in longer core vitreous removal times compared to 25G flat-tip systems (16). However, the 25G 10K cpm system effectively addressed this limitation.

This study confirmed that postoperative complications following PPV for PDR exhibit a temporal distribution and pathological heterogeneity. The intraoperative incidence of iatrogenic hemorrhage and the requirement for electrocoagulation was lower in the 25-gauge 10K cpm bevel-tip group compared with the 25-gauge flat-tip group, consistent with the mechanism of neovascular trauma during fibrovascular membrane dissection (17). Notably, early postoperative ocular hypertension frequently accompanied PPV, potentially attributable to inflammatory mediator release or tamponade agents (18), although no significant intergroup difference was observed (*p* > 0.90), perioperative intraocular pressure (IOP) monitoring in high-risk cohorts remains imperative. In contrast, the recurrent vitreous hemorrhage (VH) rate (≤6.7%) was significantly lower than literature-reported values (10–20%), a disparity potentially attributable to systematic preoperative anti-VEGF administration. Furthermore, while delayed anterior hyaloidal fibrovascular proliferation was not observed, extant literature implicates its association with sclerotomy wound management and peripheral ischemia (19), warranting consideration of adjunctive peripheral laser photocoagulation at incision sites.

Surgical safety analysis revealed no intergroup differences in sclerotomy suture requirement rates, combined cataract surgery incidence, or frequency of intraoperative pharmacologic injections (*p* > 0.05), indicating advanced instrumentation did not disrupt standard surgical workflows. Functionally, best-corrected visual acuity (BCVA) demonstrated progressive improvement throughout follow-up, with comparable gains between groups. Transient IOP elevation (1-week postoperative) did not constitute a severe adverse event. Crucially, hemorrhagic complications and endodiathermy demand showed no intergroup disparity, and severe complications were rare. Collectively, the 25-gauge beveled-tip system demonstrated equivalent efficacy and safety to conventional flat-tip instrumentation in PDR management.

Primary limitations include: (1) A restricted sample size limiting systematic comparisons (only 25-gauge flat-tip controls), hindering isolation from gauge size/cutting rate confounders; (2) absence of baseline assessment of anatomical factors (e.g., membrane adhesion severity, thickness); (3) lack of documented tamponade duration, impeding time-dependent risk analysis.

Prospective studies are warranted to validate the advantages of the beveled-tip system in PDR and elucidate the impact of different tamponades on long-term prognosis, complication profiles, and sequelae.

## Data Availability

The original contributions presented in the study are included in the article/supplementary material, further inquiries can be directed to the corresponding author.
